# SPP1 promotes cisplatin resistance in cervical cancer by regulating KRAS expression

**DOI:** 10.1371/journal.pone.0353621

**Published:** 2026-07-27

**Authors:** Changchun Long, Shiqi Wang, Yiyong Wang

**Affiliations:** Department of Obstetrics and Gynecology, Shanghai Baoshan Luodian Hospital, Baoshan, Shanghai, China; BMSCE: BMS College of Engineering, INDIA

## Abstract

**Background:**

Chemotherapy resistance is a major treatment issue for cervical cancer (CC), contributing to high morbidity and mortality. This study identifies key molecular drivers of CC progression and cisplatin (DDP) resistance, focusing on secreted phosphoprotein 1 (SPP1).

**Methods:**

Differentially expressed genes (DEGs) were identified from TCGA-CC and GSE46857 datasets. Protein-protein interaction network analysis and topological ranking determined candidate genes. SPP1 expression was evaluated in CC cell lines using qPCR and Western blotting. Cisplatin-resistant CC cells were used to examine SPP1’s role in drug resistance. Functional assays assessed cell proliferation, apoptosis, migration, and invasion.

**Results:**

Bioinformatics analysis identified SPP1 as a hub gene upregulated in CC. SPP1 expression was higher in CC and DDP-resistant cells. Silencing SPP1 inhibited cell growth and enhanced apoptosis, while overexpression had the opposite effects. Silencing SPP1 enhanced DDP sensitivity, while overexpression reduced DDP cytotoxicity. Mechanistically, SPP1 positively correlated with KRAS expression, and KRAS modulation partially reversed SPP1’s effects on DDP resistance.

**Conclusion:**

SPP1 is upregulated in CC and DDP-resistant cells, promoting tumor progression and chemoresistance via KRAS. Targeting the SPP1-KRAS axis may help overcome chemotherapy resistance in CC.

## 1. Introduction

Cervical cancer (CC) is one of the leading causes of cancer-related mortality worldwide and the fourth most common malignancy in women [1]. Epidemiological projections suggest that its incidence and mortality will increase by 56.8% and 80.7%, respectively, by 2050 [2]. The main cause of CC is a persistent infection with the high-risk human papillomavirus (HPV) [3]. The carcinogenic process is typically chronic yet preventable, often progressing asymptomatically in the early stages, which underscores the importance of routine screening in reducing mortality [4]. Current treatment strategies are determined by tumor stage, patient age, and fertility status, and primarily include surgery, concurrent chemoradiotherapy, targeted therapy, and immunotherapy [5,6]. Among chemotherapeutic agents, cisplatin remains the cornerstone of CC management [7]. However, a significant barrier to effective therapy and a major cause of treatment failure and recurrence in CC and other cancers, like ovarian cancer, is the progressive loss of tumor cell sensitivity to cisplatin after extended exposure, or even the emergence of total resistance [8,9]. Thus, elucidating the molecular mechanisms underlying cisplatin resistance in CC is of considerable clinical and research significance.

Secreted phosphoprotein 1 (SPP1) encodes osteopontin (OPN), which has a role in cell migration, adhesion, immunological modulation, and tissue repair and remodeling [10,11]. Aberrant overexpression of *SPP1* has been reported in multiple cancers, where it contributes to the tumor microenvironment through signaling pathways like PI3K/AKT and NF-κB. Specifically, in ovarian cancer, overexpression of *SPP1* has been linked to worse prognosis and greater recruitment of CD4⁺ and CD8 ⁺ T lymphocytes and macrophages [12]. Matsubara et al. identified *SPP1* as a novel marker of monocyte-derived tumor-associated macrophages (TAMs) in lung adenocarcinoma, where its high expression is associated with adverse outcomes and chemoresistance [13]. In addition, emerging evidence shows that *SPP1* is highly expressed in CC tissues and cisplatin-resistant HeLa cells (res-HeLa), and *SPP1* knockdown inhibits cell proliferation, induces apoptosis, and enhances cisplatin sensitivity by inhibiting the PI3K/AKT signaling pathway [14]. These results point to *SPP1* as a putative modulator of chemoresistance and tumor growth in CC.

Despite advances in CC treatment, cisplatin resistance remains a major challenge, leading to poor outcomes. Accumulating evidence indicates that *SPP1* not only promotes malignant phenotypes such as proliferation and invasion but also contributes to chemoresistance [15,16]. Notably, *SPP1* has been shown to regulate *KRAS* signaling in other cancers, while *KRAS* itself is implicated in cisplatin resistance [17]. However, whether *SPP1* confers cisplatin resistance in CC through *KRAS* remains unclear. To provide possible therapeutic insights for resistant CC, the goal of this study was to examine the role of *SPP1* in CC development and cisplatin resistance and ascertain if these effects are mediated via the *SPP1*-*KRAS* axis.

## 2. Materials and methods

### 2.1. Acquisition of datasets and differentially expressed genes (DEGs) screening

RNA-seq expression data for cervical cancer were obtained from The Cancer Genome Atlas Cervical Squamous Cell Carcinoma and Endocervical Adenocarcinoma project (TCGA-CESC) through the UCSC Xena Browser (https://xenabrowser.net/datapages/?dataset=TCGA.CESC.sampleMap%2FHiSeqV2&host=https%3A%2F%2Ftcga.xenahubs.net). This cohort included 306 tumor samples and 3 normal samples. Meanwhile, the GSE46857 dataset was obtained from the Gene Expression Omnibus database (https://www.ncbi.nlm.nih.gov/geo/query/acc.cgi?acc=GSE46857), including 25 cervical cancer tissue samples and 4 normal cervical tissue samples.

The R package “limma” was used to do the differential expression analysis, with *P* < 0.05 serving as the significance level and upregulated genes being defined as fold change (FC) > 2 and downregulated genes as FC < 0.5. Using the Bioinformatics and Evolutionary Genomics (https://bioinformatics.psb.ugent.be/webtools/Venn/) program, overlapping DEGs across two CC-related datasets were identified.

### 2.2. Protein-protein interaction (PPI) network construction and receiver operating characteristic (ROC) analysis of candidate genes

Overlapping DEGs were subjected to PPI analysis using Search Tool for the Retrieval of Interacting Genes (STRING, https://string-db.org/). Candidate genes were chosen by intersecting the top-ranked genes from the Maximum Neighborhood Component (MNC) and BottleNeck algorithms used to rank genes by the cytoHubba plugin in Cytoscape. Expression levels of candidate genes in CC were evaluated using the ASSISTANT for Clinical Bioinformatic platform (TCGA-CC) and Sangerbox (http://vip.sangerbox.com/home.html, GSE46857). Genes in GSE46857 were evaluated for predictive performance using ROC curves created using the R package “timeROC” (*version* 0.4). Area under the curve (AUC) values were obtained to measure diagnostic potential.

### 2.3. Cell culture and treatments

Human cervical cancer (CC) cell lines HeLa (#QS-H085), SiHa (#QS-H032), C-33A (#QS-H017), and Ca-Ski (#QS-H078), cisplatin-resistant HeLa (HeLa-DDP, #QS-Q717), as well as normal cervical epithelial cells (Ect1/E6E7, #QS-H216), were obtained from the KeyCell (Wuhan, China). All cells were maintained in RPMI-1640 medium (Gibco) containing 10% fetal bovine serum (FBS) and 1% penicillin-streptomycin at 37 °C in a humidified atmosphere with 5% CO₂. To maintain the drug-resistant phenotype, HeLa-DDP cells were kept in RPMI-1640 media with 10% FBS, 1% P/S, and 1 μg/mL DDP (Selleck) continuously. Cisplatin was initially prepared as a stock solution at 15 mg/mL in dimethylformamide (DMF). The stock solution was freshly diluted in complete medium to a working concentration of 10 μM just prior to use.

### 2.4. Cell transfections

*SPP1* overexpression plasmid (over-*SPP1*) and *KRAS* overexpression plasmid (over-*KRAS*), as well as siRNAs targeting *SPP1* (si-*SPP1*) and *KRAS* (si-*KRAS*), along with negative controls (si-NC and empty vectors), were synthesized by GenePharma (GenePharma, Shanghai, China). The si-*SPP1* sequence was 5’-CCACAAGCAGUCCAGAUUAUA-3’, reverse 5’-UAUAAUCUGGACUGCUUGUGG-3’, and the si-NC sequence was 5’-UUCUCCGAACGUGUCACGUTT-3’, reverse 5’-ACGUGACACGUUCGGAGAATT-3’. Cells were seeded in 6-well plates and cultured to reach a confluency of 60%−70%. Transfection was performed using Lipofectamine™ 2000 reagent (Invitrogen, Shanghai, China) strictly according to the manufacturer’s instructions. Cells were incubated in a humidified 37°C, 5% CO₂ environment for 6 hours post-transfection and subsequently collected for further analysis.

### 2.5. Quantitative real-time PCR (qRT-PCR)

Cellular RNA was isolated with TRIzol reagent (TaKaRa, Dalian, China) according to the supplier’s instructions. Reverse transcription was conducted using the PrimeScript RT Kit (TaKaRa, Dalian, China) to obtain cDNA. Next, using the SYBR Green Master Mix (Vazyme, Nanjing, China) and the StepOnePlus Real-Time PCR System (Applied Biosystems, China), quantitative PCR was carried out. GAPDH served as the internal reference, and gene expression levels were analyzed by the 2^−ΔΔCt^ approach. Sequences of primers were designed as follows: *SPP1*: forward 5’-CAAACGCCGACCAAGGAAAA-3’, reverse 5’-GGCCACAGCATCTGGGTATT-3’; *KRAS*: forward 5’-AGGGACTAGGGCAGTTTGGA-3’, reverse 5’-AATGTCTTGGCACACCACCA-3’; *GAPDH*: forward 5’-CATGTTGCAACCGGGAAGGA-3’, reverse 5’-ATCACCCGGAGGAGAAATCG-3’.

### 2.6. Western blotting (WB)

Protease and phosphatase inhibitors (Beyotime, Shanghai, China) were added to RIPA lysis buffer to extract total cellular proteins, and a BCA kit from the same source was used to measure the results. 30 μg of protein each sample was separated using 10% SDS-PAGE and then put onto PVDF membranes for Western blotting. Before being incubated overnight at 4 °C with primary antibodies against SPP1 (1:5000, ab181548), KRAS (1:1000, ab275876), and GAPDH (1:5000, ab8245) (all from Abcam, Shanghai, China), the membranes were blocked for one hour in 5% skim milk. After several washes, membranes were exposed to HRP-conjugated secondary antibodies (goat anti-rabbit IgG, ab6721; goat anti-mouse IgG, ab205719; 1:10000, Abcam) for 1 h at room temperature prior to detection. Protein bands were visualized using an ECL detection system (Beyotime, Shanghai, China) and quantified by ImageJ software (*version* 2.0.0).

### 2.7. Cell Counting Kit-8 (CCK-8) assay

Cell viability was assessed using the CCK-8 assay (Abcam, Shanghai, China) following the manufacturer’s protocol. Briefly, 2 × 10³ transfected CC cells were seeded in each well of a 96-well plate with 100 μL complete medium. After overnight culture, 10 μL of CCK-8 reagent was added to each well and incubated for 2 h. Absorbance at 450 nm was recorded with a Bio-Rad microplate reader (Shanghai, China) on days 1–5.

### 2.8. Apoptosis assay

Apoptosis was evaluated using the Annexin V-FITC/PI kit (BD Biosciences). For genetic manipulation, cells were transfected with siRNA or overexpression plasmids as described in Section 2.4, and apoptosis assays were performed at 24 hours post-transfection. Briefly, cells were harvested, washed twice with chilled PBS, and suspended in 1 × binding buffer at a concentration of 1 × 10^6^ cells/mL. A total of 5 μL Annexin V-FITC and 5 μL PI were added to 100 μL of suspension and incubated for 15 min at room temperature in the dark. Samples were analyzed on a FACSCanto II flow cytometer (BD Biosciences), and data were processed with FlowJo software (version 10.8.1).

### 2.9. Transwell assays

The ability of cells to migrate and invade was assessed using Transwell chambers (Corning, Shanghai, China). Two hundred and forty cells were planted into the Transwell’s top chamber for the migration test. 600 µL of media containing 10% FBS as a chemoattractant was put into the lower compartment. A cotton swab was used to gently remove the non-migrated cells after a 24-hour incubation period. For twenty minutes, migrated cells were preserved with 4% paraformaldehyde (Solarbio, Beijing, China). Cells were examined with a light microscope (Olympus, Shanghai, China) after DAPI (Beyotime, Shanghai, China) staining for 15 min. For the invasion assay, the upper chamber of the Transwell was precoated with Matrigel (Corning, Shanghai, China), and the subsequent steps were performed in the same manner as the migration assay. Migrated or invaded cells were quantified under a light microscope after staining, and the resulting counts were normalized to their respective control groups to derive the relative cell number, which served as the standardized metric for comparing migratory and invasive capacities across experimental conditions.

### 2.10. Statistical analysis

The data are presented as mean ± SD, and each experiment was independently conducted at least three times. GraphPad Prism 8.0 was used to perform statistical tests, which included the Tukey’s multiple comparison test after either the one-way ANOVA or the Student’s t-test. *P*-values less than 0.05 were regarded as statistically significant.

## 3. Results

### 3.1. Identification of SPP1 as a hub gene in CC through integrated bioinformatic analysis

**Fig 1:**
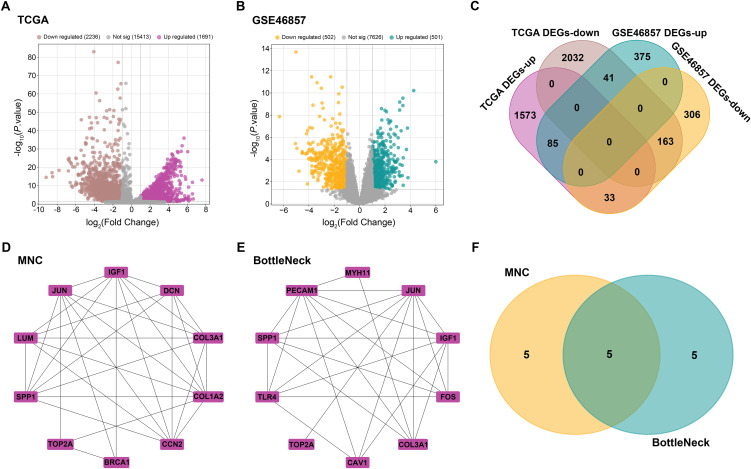
Screening and topological analysis of DEGs in CC. (A) Volcano plot showing DEGs in the TCGA-CC dataset. The X-axis represents log2 fold change (log_2_FC) and the Y-axis represents -log_10_ adjusted *P* value. Red dots indicate upregulated DEGs, brown dots indicate downregulated DEGs, and gray dots represent non-significant genes. (B) Volcano plot of DEGs in the GSE46857 dataset. Green dots indicate upregulated DEGs, and yellow dots indicate downregulated DEGs. (C) Venn diagram showing overlapping DEGs between TCGA-CC and GSE46857 datasets. (D-E) PPI network analysis of overlapping DEGs and identification of the top 10 hub genes. (D) Top 10 genes ranked by the MNC algorithm; (E) Top 10 genes ranked by the BottleNeck algorithm. (F) Venn diagram showing five candidate genes identified by overlapping the top 10 genes from the MNC and BottleNeck algorithms. DEGs: differentially expressed genes; TCGA: The Cancer Genome Atlas; CC: cervical cancer; PPI: protein-protein interaction; MNC: Maximum Neighborhood Component.

**Fig 2:**
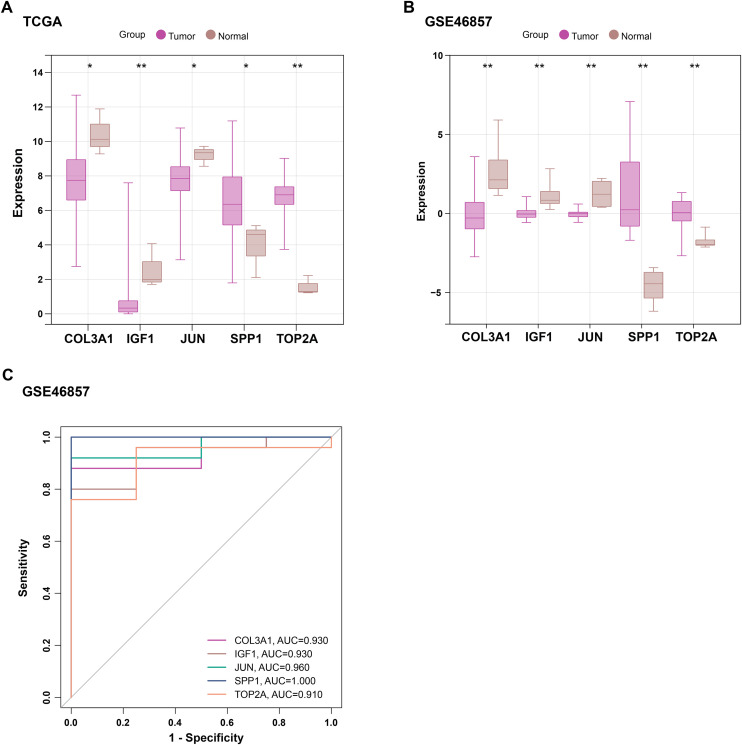
Expression validation and predictive performance of candidate hub genes in CC datasets. (A) Boxplots showing the expression levels of five candidate genes in tumor versus normal cervical tissues in the TCGA-CC dataset. The Y-axis represents expression, and the X-axis indicates sample groups (Tumor, Normal). Group comparisons were performed using the Mann-Whitney U test. (B) Boxplots showing the expression levels of five candidate genes in tumor versus normal cervical tissues in the GSE46857 dataset. Group comparisons were performed using the Mann-Whitney U test. (C) ROC curve analysis of five candidate genes in the GSE46857 dataset. The X-axis represents the false positive rate (1-specificity) and the Y-axis represents the true positive rate (sensitivity). The AUC quantifies the predictive performance. CC: cervical cancer; ROC: receiver operating characteristic; AUC: area under the curve. *P < 0.05, **P < 0.01.

Using the TCGA-CC dataset, a total of 2,236 downregulated and 1,691 upregulated DEGs were identified (Fig 1A). Similarly, analysis of the GSE46857 dataset yielded 502 downregulated and 501 upregulated DEGs (Fig 1B). Topological comparison of DEGs from both datasets revealed 248 overlapping genes (Fig 1C). The top ten most linked genes in each method were determined by ranking these overlapping DEGs using the MNC and BottleNeck algorithms after a subsequent PPI network analysis (Figs 1D and 1E). Because these two algorithms capture different topological characteristics, only genes that were simultaneously included in the top ten of both methods were considered robust. A Venn intersection of the two top ten gene sets identified five consistently high-ranking candidates: *COL3A1*, *IGF1*, *SPP1*, *JUN*, and *TOP2A* (Fig 1F). Expression profiling of these candidates in tumor and normal samples showed that *SPP1* and *TOP2A* were consistently upregulated in tumors across both TCGA-CC and GSE46857 datasets, whereas *COL3A1*, *IGF1*, and *JUN* were downregulated (Figs 2A and 2B). ROC curve analysis further evaluated the predictive value of these candidates in the GSE46857 dataset, with *SPP1* exhibiting excellent predictive performance (AUC = 1), thereby selected as the hub gene for subsequent analyses (Fig 2C).

### 3.2. SPP1 enhances the malignant phenotype of CC cells

**Fig 3:**
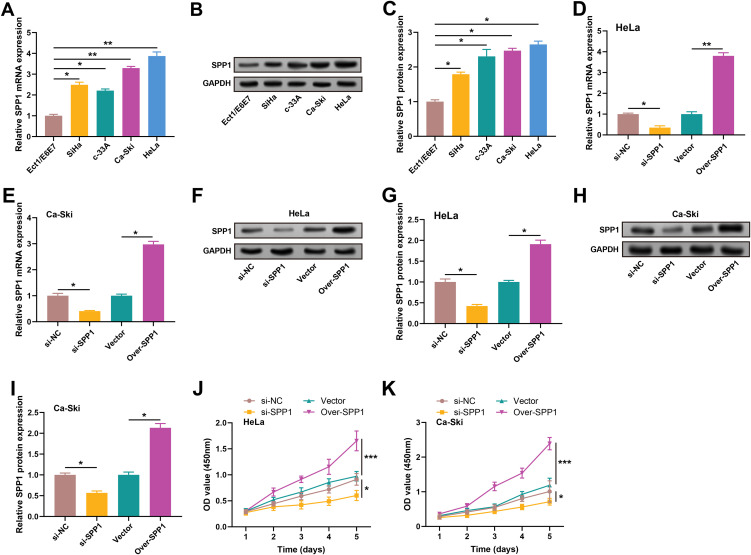
Expression and modulation of *SPP1* in CC cell lines. (A) Relative *SPP1* mRNA levels in normal cervical epithelial cells (Ect1/E6E7) and cervical cancer cell lines (SiHa, C-33A, Ca-Ski, HeLa) measured by qRT-PCR. The Y-axis represents relative *SPP1* expression normalized to GAPDH, and the X-axis represents cell lines. Statistical analysis was performed using one-way ANOVA. (B-C) *SPP1* protein expression was detected by WB in the same cell lines. Quantification of SPP1 protein levels normalized to GAPDH (C). Statistical analysis was performed using one-way ANOVA. (D-E) Relative *SPP1* mRNA levels in HeLa (D) and Ca-Ski (E) cells after transfection with si-*SPP1* or over-*SPP1* measured by qRT-PCR. Statistical analysis was performed using one-way ANOVA. (F-I) SPP1 protein levels in HeLa (F, G) and Ca-Ski (H, I) cells after si-*SPP1* or over-*SPP1* transfection were measured by WB. Statistical analysis was performed using one-way ANOVA. (J-K) CCK-8 assays were performed to assess cell proliferation in HeLa (J) and Ca-Ski (K) cells following si-*SPP1* or over-*SPP1* transfection. The Y-axis represents OD value (450nm), and the X-axis represents time points (days). Statistical analysis was performed using two-way ANOVA. CC: cervical cancer; qRT-PCR: quantitative real-time polymerase chain reaction; WB: Western blotting; CCK-8: Cell Counting Kit-8. *P < 0.05, **P < 0.01. Data are presented as mean ± standard deviation (SD) from three independent experiments (n = 3).

**Fig 4:**
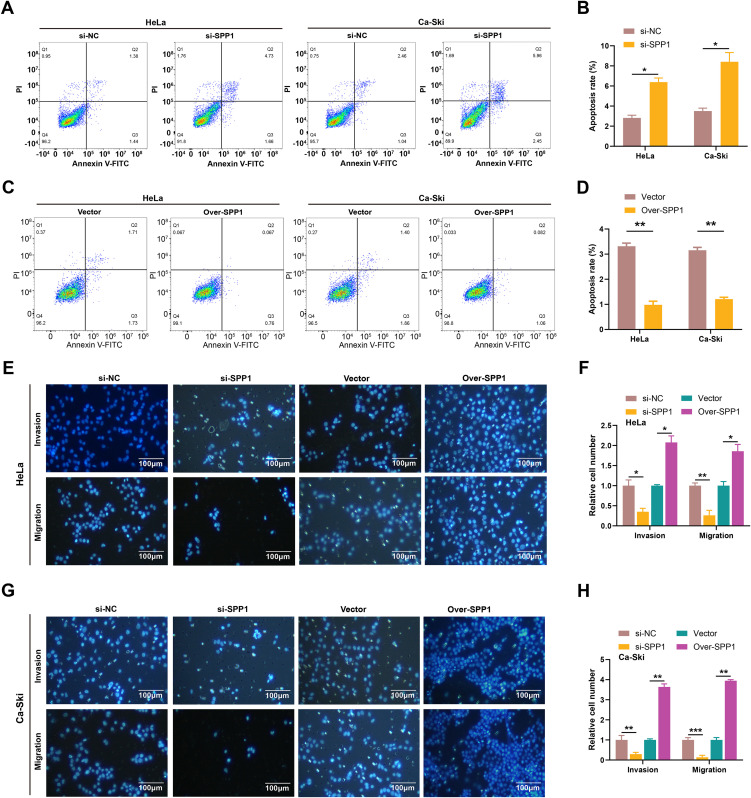
Functional effects of *SPP1* on proliferation, apoptosis, migration, and invasion of CC cells. (A-B) Flow cytometric analysis of apoptosis in HeLa and Ca-Ski cells transfected with si-NC or si-*SPP1*. Quantification of apoptotic cell populations is shown in panel B. Quadrants: Q1 (Top Left): Necrotic cells (Annexin V − /PI+); Q2 (Top Right): Late apoptotic cells (Annexin V + /PI+); Q3 (Bottom Right): Early apoptotic cells (Annexin V + /PI−); Q4 (Bottom Left): Live cells (Annexin V − /PI−). Total apoptosis was calculated as the sum of early and late apoptotic populations (Q2 + Q3). Statistical analysis was performed using an unpaired Student’s t-test. (C-D) Flow cytometric analysis of apoptosis in HeLa and Ca-Ski cells transfected with vector control or over-*SPP1* plasmid. Quantification of apoptotic cell populations is shown in panel D. Quadrants: Q1 (Top Left): Necrotic cells (Annexin V − /PI+); Q2 (Top Right): Late apoptotic cells (Annexin V + /PI+); Q3 (Bottom Right): Early apoptotic cells (Annexin V + /PI−); Q4 (Bottom Left): Live cells (Annexin V − /PI−). Total apoptosis was calculated as the sum of early and late apoptotic populations (Q2 + Q3). Statistical analysis was performed using an unpaired Student’s t-test. (E-F) Transwell invasion and migration assays in HeLa cells following *SPP1* knockdown or overexpression. Representative images are shown in panel E, with quantitative analysis in panel F. Statistical analysis was performed using one-way ANOVA. (G-H) Transwell invasion and migration assays in Ca-Ski cells following *SPP1* knockdown or overexpression. Representative images are shown in panel G, with quantitative analysis in panel H. Statistical analysis was performed using one-way ANOVA. CC: cervical cancer. *P < 0.05, **P < 0.01, ***P < 0.001. Data are presented as mean ± standard deviation (SD) from three independent experiments (n = 3).

The expression profile of *SPP1* was assessed in Ect1/E6E7 and four CC cell lines using qRT-PCR and WB. Both assays consistently demonstrated markedly higher *SPP1* expression in all cancer cell lines relative to normal controls (Figs 3A-3C). Among the cancer cell lines, HeLa and SiHa displayed the most pronounced upregulation and were consequently chosen for subsequent functional experiments. To verify the efficacy of *SPP1* modulation, HeLa and Ca-Ski cells were transfected with either siRNA targeting *SPP1* (si-*SPP1*) or an *SPP1* overexpression plasmid (over-*SPP1*). qRT-PCR and WB showed decreased SPP1 after si-*SPP1* and increased expression after over-*SPP1* transfection (Figs 3D-3I). *SPP1* overexpression increased proliferative ability, whereas *SPP1* knockdown significantly inhibited the proliferation of HeLa and Ca-Ski cells, according to CCK-8 analysis (Figs 3J and 3K). Further investigation using flow cytometry showed that silencing *SPP1* significantly increased apoptosis, while overexpression reduced apoptotic cell fractions (Figs 4A-4D). Transwell experiments demonstrated that SPP1 suppression significantly reduced the migration and invasion capacities of CC cells, whereas SPP1 overexpression markedly increased these abilities in both HeLa and Ca-Ski cell lines (Figs 4E-4H).Together, these results demonstrate that *SPP1* functions as a pro-tumorigenic factor in CC by promoting proliferation, migration, and invasion while suppressing apoptosis.

### 3.3. SPP1 reduces the sensitivity of CC cells to DDP

**Fig 5:**
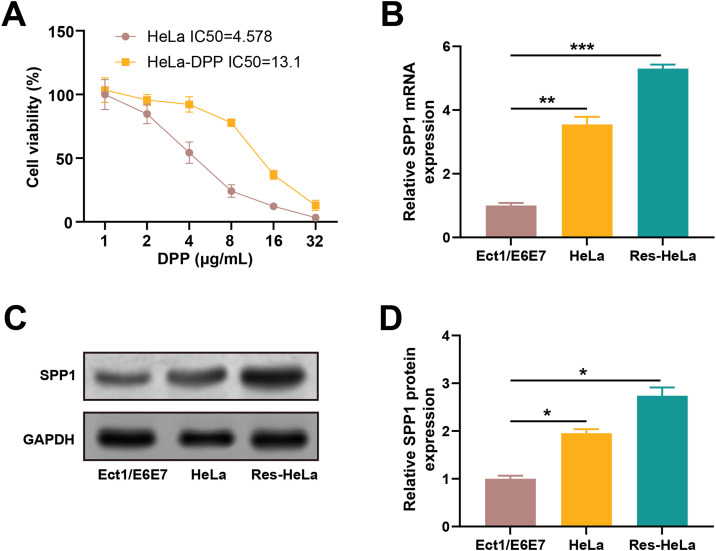
Progressive upregulation of *SPP1* in DDP-resistant CC cells. (A) Cell viability curves of HeLa and Res-HeLa cells after treatment with increasing concentrations of DDP, measured by CCK-8 assay. The IC50 values were calculated to confirm the drug-resistant phenotype. (B) Relative *SPP1* mRNA levels in Ect1/E6E7, HeLa, and Res-HeLa cells detected by qRT-PCR. Y-axis: relative expression normalized to GAPDH; X-axis: cell lines. Statistical analysis was performed using one-way ANOVA. (C-D) *SPP1* protein levels in the same cells were measured by WB. (C) Representative WB images; (D) Quantification normalized to GAPDH. Statistical analysis was performed using one-way ANOVA.Y-axis: relative protein expression; X-axis: cell lines. DDP: cisplatin; CC: cervical cancer; CCK-8: Cell Counting Kit-8; IC50: half-maximal inhibitory concentration; qRT-PCR: quantitative real-time polymerase chain reaction; WB: Western blotting. *P < 0.05, **P < 0.01. Data are presented as mean ± standard deviation (SD) from three independent experiments (n = 3).

**Fig 6:**
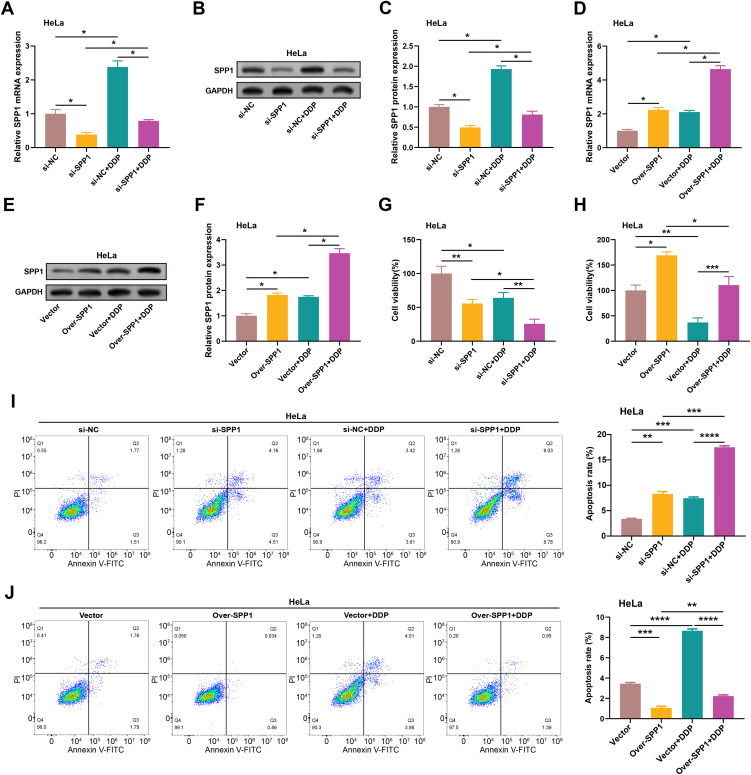
Effects of *SPP1* modulation on cisplatin sensitivity in HeLa cells. (A) *SPP1* mRNA levels in HeLa cells after si-*SPP1* and/or DDP treatment measured by qRT-PCR. Statistical analysis was performed using one-way ANOVA. (B-C) SPP1 protein levels were detected by WB in the same groups. Quantification normalized to GAPDH (C). Statistical analysis was performed using one-way ANOVA. (D) *SPP1* mRNA levels in HeLa cells after overexpression of *SPP1* and/or DDP treatment were measured by qRT-PCR. Statistical analysis was performed using one-way ANOVA. (E-F) SPP1 protein levels were detected by WB in the same groups. Quantification normalized to GAPDH (F). Statistical analysis was performed using one-way ANOVA. (G) Cell viability of HeLa cells treated with si-*SPP1* and/or DDP was measured by CCK-8. Y-axis: relative viability; X-axis: experimental groups. Statistical analysis was performed using one-way ANOVA. (H) Cell viability of HeLa cells treated with over-*SPP1* and/or DDP was measured by CCK-8. Statistical analysis was performed using one-way ANOVA. (I) Apoptosis in HeLa cells treated with si-*SPP1* and/or DDP assessed by flow cytometry. Y-axis: apoptosis rate (%); X-axis: experimental groups. Quadrants: Q1 (Top Left): Necrotic cells (Annexin V − /PI+); Q2 (Top Right): Late apoptotic cells (Annexin V + /PI+); Q3 (Bottom Right): Early apoptotic cells (Annexin V + /PI−); Q4 (Bottom Left): Live cells (Annexin V − /PI−). Total apoptosis was calculated as the sum of early and late apoptotic populations (Q2 + Q3).Statistical analysis was performed using one-way ANOVA. (J) Apoptosis in HeLa cells treated with over-*SPP1* and/or DDP was assessed by flow cytometry. Quadrants: Q1 (Top Left): Necrotic cells (Annexin V − /PI+); Q2 (Top Right): Late apoptotic cells (Annexin V + /PI+); Q3 (Bottom Right): Early apoptotic cells (Annexin V + /PI−); Q4 (Bottom Left): Live cells (Annexin V − /PI−). Total apoptosis was calculated as the sum of early and late apoptotic populations (Q2 + Q3). Statistical analysis was performed using one-way ANOVA. DDP: cisplatin; qRT-PCR: quantitative real-time polymerase chain reaction; WB: Western blotting; CCK-8: Cell Counting Kit-8. *P < 0.05, **P < 0.01, ***P < 0.001. Data are presented as mean ± standard deviation (SD) from three independent experiments (n = 3).

Comparison of cisplatin sensitivity demonstrated a markedly elevated IC50 in DDP-resistant HeLa cells (Res-HeLa) relative to parental HeLa cells (Fig 5A), confirming the resistant phenotype. qRT-PCR and WB investigations revealed that SPP1 expression was considerably higher in HeLa cells compared to Ect1/E6E7 cells, and much higher in Res-HeLa (Figs 5B-5D). These results may suggest a progressive upregulation of *SPP1* is associated with malignant transformation and the acquisition of DDP resistance. To evaluate the functional role of *SPP1* in the response to DDP, HeLa cells were transfected with si-*SPP1* or an *SPP1* overexpression plasmid and then treated with DDP. Silencing *SPP1* markedly reduced its expression and attenuated the DDP-induced upregulation (Figs 6A-6C), whereas overexpression alone enhanced *SPP1* levels and synergized with DDP to further increase protein expression (Figs 6D-6F). Functionally, CCK-8 assays revealed that *SPP1* knockdown inhibited cell viability, and this effect was enhanced in combination with DDP, while overexpression promoted cell viability and counteracted the inhibitory effect of DDP (Figs 6G-6H). Consistently, flow cytometry analysis demonstrated that apoptosis was increased by si-*SPP1* or DDP treatment, with the strongest induction observed under combined treatment. Conversely, *SPP1* overexpression reduced apoptosis and mitigated the pro-apoptotic effect of DDP (Figs 6I-6J). Together, these findings demonstrate that *SPP1* is progressively upregulated in cisplatin-resistant CC cells and contributes to chemoresistance by sustaining proliferation and suppressing apoptosis.

### 3.4. SPP1 modulates KRAS expression and mediates its response to DDP in CC cells

**Fig 7:**
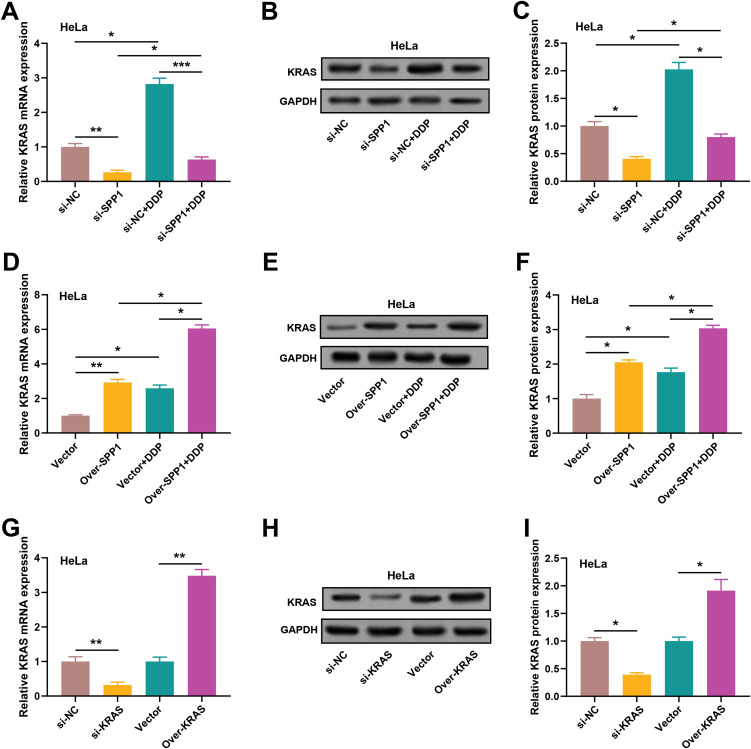
*SPP1* regulates *KRAS* expression and modulates its response to DDP. (A) *KRAS* mRNA levels in HeLa cells after si-*SPP1* and/or DDP treatment measured by qRT-PCR. Y-axis: relative mRNA expression; X-axis: experimental groups. Statistical analysis was performed using one-way ANOVA. (B-C) *KRAS* protein levels in the same cells were detected by WB. Quantification of protein expression normalized to GAPDH (C). Statistical analysis was performed using one-way ANOVA. (D) *KRAS* mRNA levels in HeLa cells after overexpression of *SPP1* and/or DDP treatment were measured by qRT-PCR. Statistical analysis was performed using one-way ANOVA. (E-F) KRAS protein levels in the same cells were detected by WB. Quantification normalized to GAPDH (F). Statistical analysis was performed using one-way ANOVA. (G) *KRAS* mRNA levels in HeLa cells transfected with si-*KRAS* or over-*KRAS* were measured by qRT-PCR. Statistical analysis was performed using one-way ANOVA. (H-I) KRAS protein levels in the same cells were detected by WB. Quantification normalized to GAPDH (I). Statistical analysis was performed using one-way ANOVA. DDP: cisplatin; qRT-PCR: quantitative real-time polymerase chain reaction; WB: Western blotting. *P < 0.05, **P < 0.01, ***P < 0.001. Data are presented as mean ± standard deviation (SD) from three independent experiments (n = 3).

A previous study has suggested that *SPP1* can induce DDP resistance in CC [14], but the specific mechanism remains unclear. Given that *SPP1* can induce drug resistance in head and neck cancer by activating the *KRAS/MEK* pathway [17], and that *KRAS* has been linked to DDP resistance in nasopharyngeal carcinoma [18], we hypothesized that *KRAS* may be a key node in *SPP1*-mediated cisplatin resistance in CC. To test this hypothesis, we interfered with *SPP1* in HeLa cells and measured *KRAS* levels. qRT-PCR and WB analysis showed that si-*SPP1* significantly downregulated *KRAS*, while DDP alone upregulated KRAS; si-*SPP1* plus DDP partially reversed the DDP-induced *KRAS* increase (Figs 7A-7C). Conversely, *SPP1* overexpression or DDP alone increased *KRAS* expression, and the combined effect was further enhanced (Figs 7D-7F). Transfection efficiency was confirmed, showing reduced *KRAS* expression with si-*KRAS* and increased expression with over-*KRAS* (Figs 7G-7I). These results may suggest that *SPP1* positively regulates *KRAS* expression in CC cells and modulates their response to DDP treatment.

### 3.5. KRAS mediates the effects of SPP1 on DDP sensitivity in CC cells

**Fig 8:**
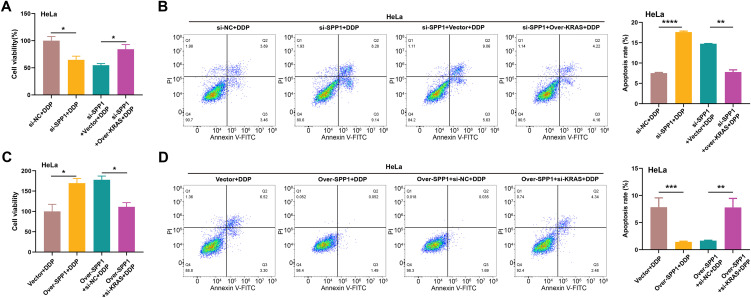
*SPP1* enhances cisplatin resistance via *KRAS* signaling. (A) Cell viability in HeLa cells treated with 10 µM DDP following si-*SPP1* and/or over-*KRAS* transfection measured by CCK-8. Y-axis: relative viability; X-axis: experimental groups. Statistical analysis was performed using one-way ANOVA. (B) Apoptosis in HeLa cells treated with 10 µM DDP and transfected with si-*SPP1* and/or over-*KRAS* was measured by flow cytometry. Y-axis: apoptosis rate (%); X-axis: experimental groups. Quadrants: Q1 (Top Left): Necrotic cells (Annexin V − /PI+); Q2 (Top Right): Late apoptotic cells (Annexin V + /PI+); Q3 (Bottom Right): Early apoptotic cells (Annexin V + /PI−); Q4 (Bottom Left): Live cells (Annexin V − /PI−). Total apoptosis was calculated as the sum of early and late apoptotic populations (Q2 + Q3).Statistical analysis was performed using one-way ANOVA. (C) Cell viability in HeLa cells treated with 10 µM DDP following over-*SPP1* and/or si-*KRAS* transfection was detected by CCK-8. Statistical analysis was performed using one-way ANOVA. (D) Apoptosis in HeLa cells treated with 10 µM DDP and transfected with over-*SPP1* and/or si-*KRAS* was measured by flow cytometry. Quadrants: Q1 (Top Left): Necrotic cells (Annexin V − /PI+); Q2 (Top Right): Late apoptotic cells (Annexin V + /PI+); Q3 (Bottom Right): Early apoptotic cells (Annexin V + /PI−); Q4 (Bottom Left): Live cells (Annexin V − /PI−). Total apoptosis was calculated as the sum of early and late apoptotic populations (Q2 + Q3). Statistical analysis was performed using one-way ANOVA. DDP: cisplatin; CCK-8: cell counting kit-8. *P < 0.05, **P < 0.01, ***P < 0.001. Data are presented as mean ± standard deviation (SD) from three independent experiments (n = 3).

To further determine whether *KRAS* is required for *SPP1*-mediated DDP resistance, rescue experiments were performed in HeLa cells. CCK-8 assays showed that si-SPP1 significantly reduced cell viability under DDP treatment, while co-transfection with an overexpression plasmid of *KRAS* (over-*KRAS*) partially restored cell viability (Fig 8A). Consistently, *SPP1* silencing markedly enhanced DDP-induced apoptosis, whereas *KRAS* overexpression reversed this effect, reducing apoptotic cell populations (Fig 8B). Conversely, *SPP1* overexpression promoted cell viability and suppressed apoptosis under DDP exposure, but these effects were attenuated by *KRAS* knockdown (Figs 8C and 8D). Collectively, these findings indicate that *KRAS* may serve as a potential downstream effector of *SPP1*, possibly participating in regulating cell survival and apoptosis under DDP treatment.

## 4. Discussion

We identified *SPP1* as a hub gene in CC using an integrated bioinformatic analysis of several transcriptome datasets. *SPP1* was consistently upregulated in both clinical samples and CC cell lines. Functional experiments revealed that *SPP1* increases cell growth while inhibiting apoptosis, emphasizing its multimodal pro-tumorigenic activity. Notably, *SPP1* expression increased in DDP-resistant cells, suggesting a direct association with chemoresistance. Mechanistic investigations revealed that *SPP1* modulates *KRAS* expression and influences its response to DDP, thereby mediating cellular sensitivity to chemotherapy. Manipulation of *SPP1* levels significantly altered cell viability and apoptotic rates under DDP treatment, and these effects were partially rescued or reversed through corresponding modulation of *KRAS*, indicating a functional *SPP1*-*KRAS* signaling axis in conferring drug resistance. Collectively, these results highlight *SPP1* as a potential therapeutic target and suggest that interventions targeting the *SPP1*-*KRAS* pathway could improve the efficacy of conventional chemotherapy in resistant cervical tumors.

Through integrated bioinformatic analyses of multiple CC-related datasets, five differentially expressed and highly correlated candidate genes (*COL3A1*, *IGF1*, *SPP1*, *JUN*, and *TOP2A*) were identified. Previous studies have implicated several of these genes in CC progression. Lu et al.[19] reported that miR-186-3p directly targets *IGF1* mRNA, downregulating its expression and thereby inhibiting PI3K/AKT pathway activation, which suppresses CC cell proliferation and invasion while promoting apoptosis. Sun et al.[20] demonstrated that a subset of epithelial cells with elevated *TOP2A* expression exhibits high proliferative and metabolic activity, along with matrix plasticity, and modulates the tumor microenvironment through the *LAMC1*-(*ITGA3*-*ITGB1*) axis. Additionally, Xia et al.[21] found significant enrichment of *SPP1*-positive macrophages in cervical tumors, which interact with immune cells via the *SPP1*-*CD44* signaling axis to establish an immunosuppressive microenvironment that facilitates disease progression. To further assess the clinical relevance of these candidates, ROC curve analyses were performed, revealing that *SPP1* displayed the highest diagnostic potential (AUC = 1), supporting its selection as the hub gene for subsequent functional and mechanistic studies.

Cisplatin, a platinum-based chemotherapeutic agent, exerts cytotoxicity by forming DNA adducts, inducing DNA damage, replication blockade, and apoptosis [22,23]. However, prolonged cisplatin treatment often leads to chemoresistance, severely limiting therapeutic efficacy [24]. Previous investigations have explored strategies to overcome cisplatin resistance in CC. Bhattacharjee et al.[25] systematically analyzed resistance mechanisms and proposed combinatorial approaches involving miRNAs, CRISPR/Cas systems, and cisplatin-based therapies to enhance anticancer effects. Pasha et al.[26] similarly generated a cisplatin-resistant HeLa model and demonstrated that Andrographolide, a natural compound, acts synergistically with cisplatin to inhibit cell growth and motility while inducing apoptosis. This effect involved downregulation of *SPP1* and NF-κB, inhibition of the PI3K/AKT pathway, and upregulation of *PTEN*, suggesting potential therapeutic strategies for resistant CC. Consistent with these findings, our results revealed progressively elevated *SPP1* expression from normal cervical cells to parental HeLa cells and further in cisplatin-resistant HeLa cells, implicating *SPP1* upregulation as a critical contributor to both cervical carcinogenesis and acquired cisplatin resistance.

*KRAS* encodes a GTPase of the RAS family, and activating mutations in *KRAS* result in constitutive signaling that persistently stimulates downstream pathways, including MAPK/ERK and PI3K/AKT, thereby promoting tumor initiation and progression [27,28]. Previous studies have implicated the *SPP1*-*KRAS* axis in cancer development and therapy resistance. According to Liu et al.[17], *SPP1* is extensively expressed in HNSCC tissues and mediates malignant progression and cetuximab resistance through activation of the *KRAS*/MEK pathway, whereas *SPP1* knockdown or MEK inhibition effectively reversed resistance. Wang et al.[29] demonstrated that ferroptosis occurs in high-risk HPV-induced cervical intraepithelial lesions but is suppressed in cervical squamous cell carcinoma, with sustained ferroptosis influencing *KRAS* expression and contributing to malignant transformation. Similarly, *GPX2* was found to be upregulated in *KRAS*-driven NSCLC, promoting tumor progression and cisplatin resistance; its inhibition provided a potential therapeutic avenue [30]. In line with these results, we observed that silencing *SPP1* decreased *KRAS* expression and sensitized cells to cisplatin-induced apoptosis, while its overexpression upregulated *KRAS* and reduced apoptosis, ultimately favoring cell survival. Furthermore, modulating *KRAS* expression partially reversed the effects of *SPP1* on cisplatin sensitivity, indicating that *SPP1* might regulate chemoresistance in CC at least partly through *KRAS* signaling.

This study has several limitations of different types. Regarding data and methodology, the numbers of tumor and normal samples in the TCGA dataset are imbalanced, and the ROC analysis was based solely on a single small‑sample GEO dataset, with insufficient systematic integration of clinical stratification information. In terms of external validation, validation using independent databases and clinical tissue samples is lacking. As for mechanistic exploration, the specific downstream *KRAS* pathways were not deeply investigated. Consequently, the conclusions of this study require further in *vivo* and clinical validation. Future work will incorporate additional independent cervical cancer GEO datasets, TCGA cohorts, and clinical samples, as well as multicenter cohorts, stage‑stratified samples, and in *vivo* experiments, to enhance the robustness and reliability of the findings and to further support and refine the current conclusions.

## 5. Conclusion

In conclusion, this study identifies *SPP1* as a key hub gene in CC and demonstrates its pivotal role in tumor progression and cisplatin resistance. Bioinformatic analyses confirmed its consistent upregulation in CC tissues, suggesting its potential as a candidate biomarker. Functional experiments showed that *SPP1* enhances cell growth and motility while reducing apoptosis, with higher expression observed in cisplatin-resistant cells. Mechanistically, *SPP1* positively regulates *KRAS*, and modulation of *KRAS* partially reverses *SPP1*-mediated effects on drug sensitivity, highlighting the *SPP1*-*KRAS* axis as a driver of chemoresistance. These findings provide novel insights into CC pathogenesis and suggest that targeting *SPP1* or its downstream *KRAS* signaling may represent a potential therapeutic strategy to overcome cisplatin resistance.

## Supporting information

S1 Data WB-Raw data. Original Western blot images for SPP1, KRAS, and GAPDH corresponding to Figures 3B, 3F, 3H, 5C, 6B, 6E, 7B, 7E, and 7H. GAPDH was used as the loading control.
(PDF)
