# Supplementary material for: SPP1 promotes cisplatin resistance in cervical cancer by regulating KRAS expression
Source: PLoS One. 2026 Jul 27;21(7):e0353621. doi: 10.1371/journal.pone.0353621 (PMC13405077; doi:10.1371/journal.pone.0353621)
Supplement: S1 Data — Original Western blot images for SPP1, KRAS, and GAPDH corresponding to Figures 3B, 3F, 3H, 5C, 6B, 6E, 7B, 7E, and 7H. GAPDH was used as the loading control. (PDF) [file pone.0353621.s001.pdf]

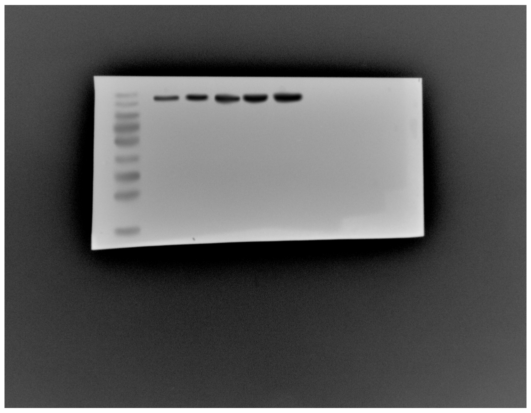
Figure 3B_SPP1

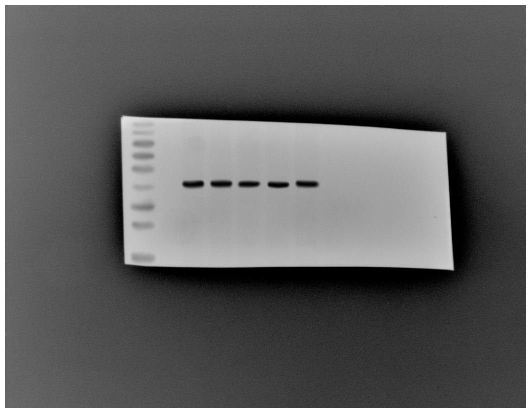
Figure 3B_GAPDH

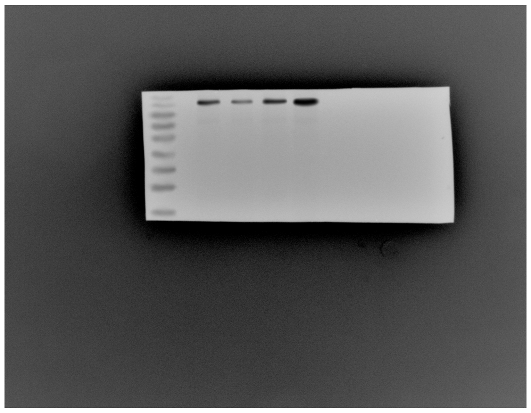
Figure 3F_SPP1_HeLa

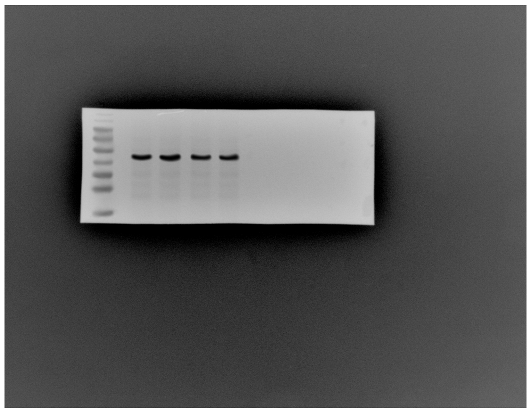
Figure 3F_GAPDH_HeLa

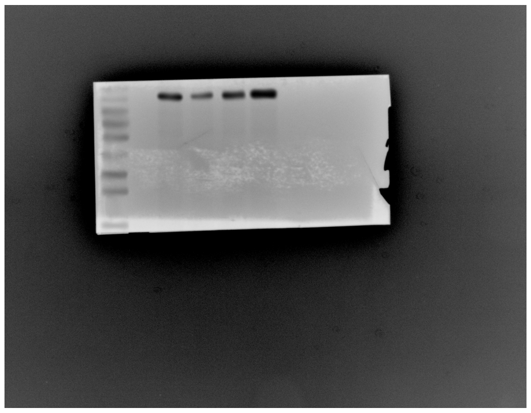
Figure 3H_SPP1_Ca-Ski

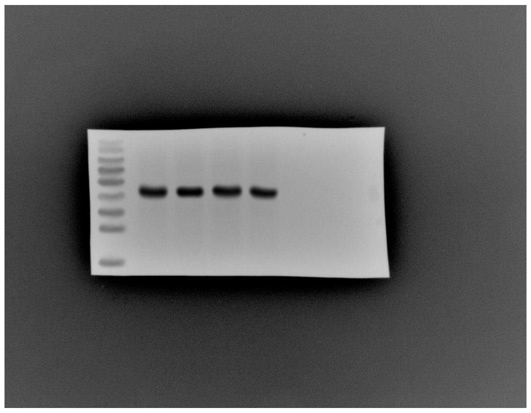
Figure 3H_GAPDH_Ca-Ski

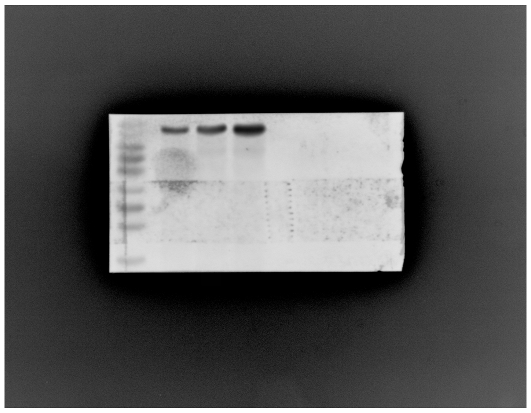
Figure 5C_SPP1

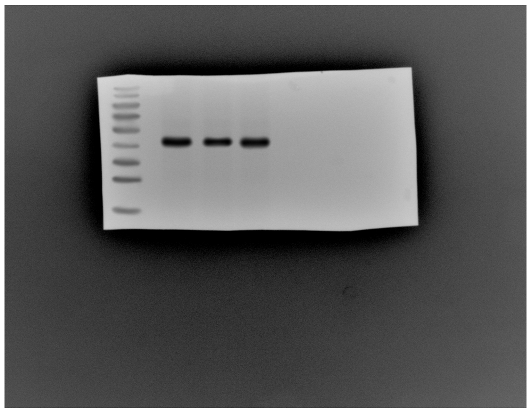
Figure 5C_GAPDH

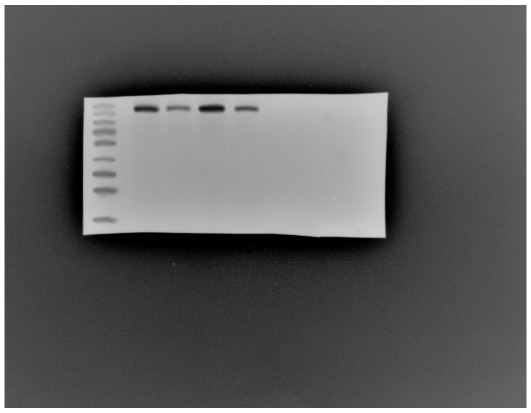
Figure 6B_SPP1

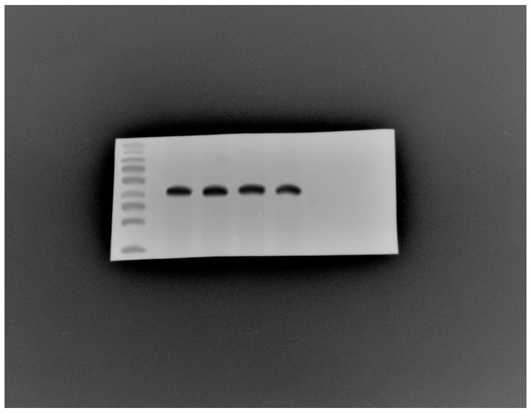
Figure 6B_GAPDH

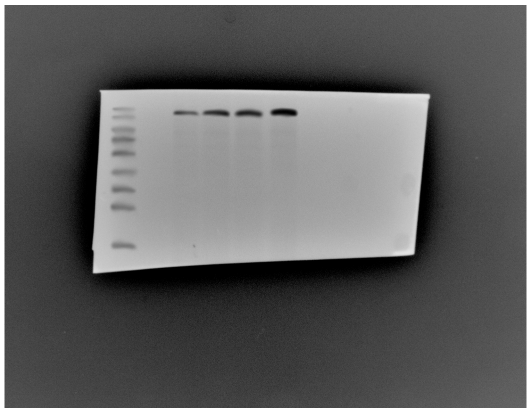
Figure 6E_SPP1

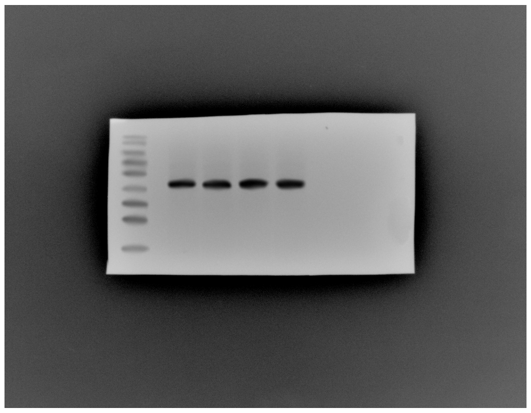
Figure 6E_GAPDH

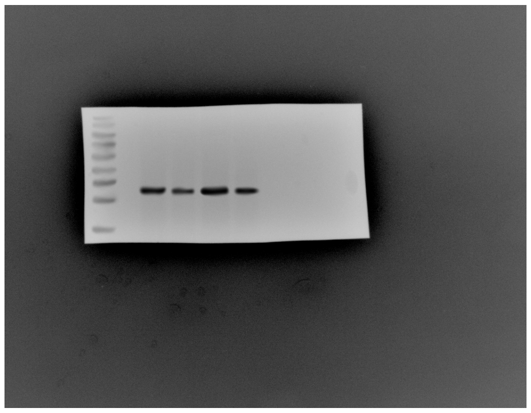
Figure 7B_KRAS

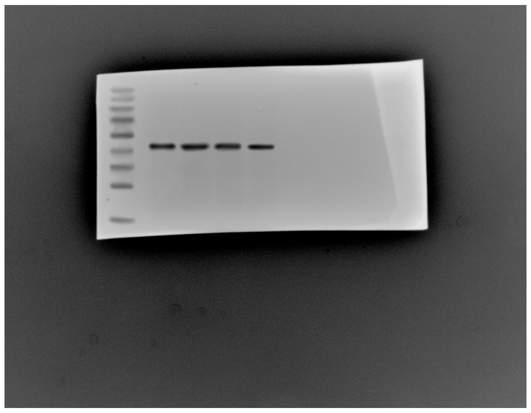
Figure 7B_GAPDH

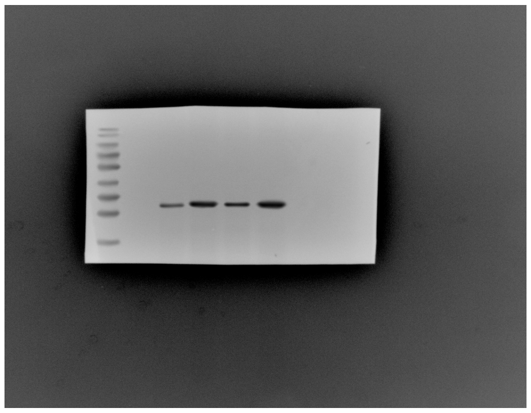
Figure 7E_KRAS

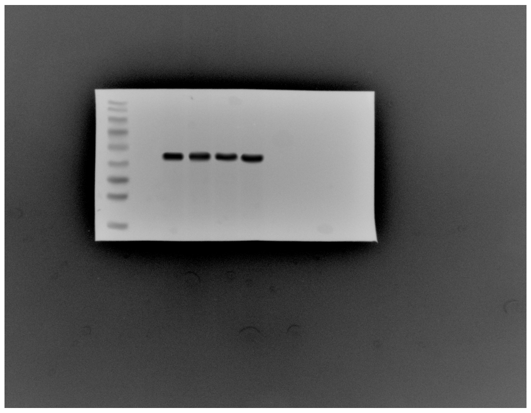
Figure 7E_GAPDH

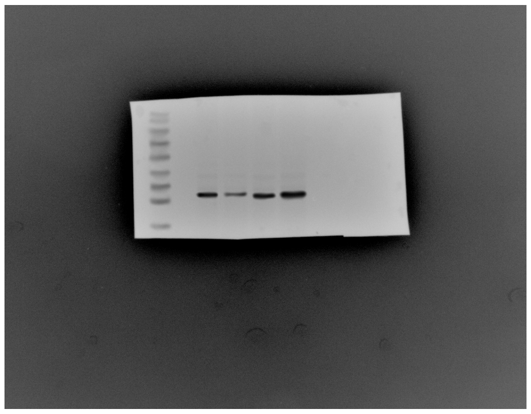
Figure 7H_KRAS

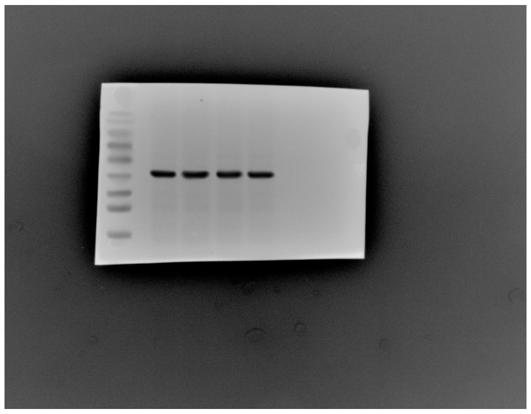
Figure 7H_GAPDH
